# Seneca Valley virus 3C protease targets the Nrf2/HO-1 pathway to antagonize its antiviral activity

**DOI:** 10.1128/jvi.01656-25

**Published:** 2026-01-05

**Authors:** Jiangwei Song, Teng Liu, Jingjing Yang, Liwei Zhao, Jiayao Su, Zijian Li, Ruiyi Ma, Xuexia Wen, Peipei Cheng

**Affiliations:** 1 Beijing Key Laboratory for Prevention and Control of Infectious Diseases in Livestock and Poultry, Institute of Animal Husbandry and Veterinary Medicine, Beijing Academy of Agriculture and Forestry Sciences 107624, Beijing, China; 2 The Platform of Youth Innovation, Institute of Animal Husbandry and Veterinary Medicine, Beijing Academy of Agricultural and Forestry Sciences, Beijing, China; 3 College of Animal Science and Veterinary Medicine, Shenyang Agricultural University 98428, Shenyang, China; 4 Beijing Biomedicine Technology Center, Zhaofeng Hua Biotechnology, Beijing, China; University of Michigan Medical School, Ann Arbor, Michigan, USA

**Keywords:** Seneca Valley virus (SVV), nuclear factor erythroid 2-related factor 2 (Nrf2), heme oxygenase-1 (HO-1), 3C protease

## Abstract

Seneca Valley virus (SVV) infection gives rise to severe vesicular diseases in pigs, presenting a substantial threat to the global swine industry. The redox imbalance resulting from oxidative stress is an essential pathogenic mechanism during viral infections. Nevertheless, the regulatory mechanisms of oxidative stress by viral and host factors during SVV infection remain elusive. In this study, we discovered that SVV elicited cellular oxidative stress through the induction of reactive oxygen species production and the suppression of the nuclear factor erythroid 2-related factor 2 (Nrf2)/heme oxygenase-1 (HO-1) pathway. Our findings indicated that the overexpression of Nrf2/HO-1 exerted a remarkable anti-SVV effect. Conversely, the inhibition of Nrf2/HO-1 expression facilitated the proliferation of SVV. HO-1 metabolic products carbon monoxide and biliverdin inhibit SVV replication. HO-1 promotes type I interferon response and interferon-stimulated gene expressions, which contribute to its antiviral mechanism. Furthermore, our findings reveal that the SVV 3C proteinase targets the Nrf2/HO-1 axis for degradation via caspase pathway, thereby promoting viral replication. Collectively, these results clarify the convoluted molecular mechanisms by which SVV weakens the host’s antioxidant defense system and suggest potential targets for therapeutic interventions regarding SVV infections.

**IMPORTANCE:**

Nrf2 is a crucial redox regulator responsible for initiating the expression of downstream antioxidant genes, including HO-1 and superoxide dismutase. HO-1, an enzyme induced by stress, performs protective roles through the conversion of heme into carbon monoxide, biliverdin, and iron. Nevertheless, the function of Nrf2/HO-1 during Seneca Valley virus (SVV) infection is yet to be clearly defined. In this study, we showed that SVV infection led to a reduction in the expression of Nrf2/HO-1, and the overexpression of Nrf2/HO-1 induced a potent anti-SVV effect. SVV 3C proteinase promoted the caspase-dependent degradation of Nrf2/HO-1. As a result, it attenuated the cell's ability to resist oxidative stress and counteracted the antiviral function of Nrf2/HO-1. Our research further uncovered a novel mechanism through which SVV eludes the host's antiviral effects by disrupting cellular redox balance, offering important targets for preventing and controlling SVV infection.

## INTRODUCTION

Seneca Valley virus (SVV) infection causes vesicular disease in pigs, which is clinically indistinguishable from other highly pathogenic porcine vesicular diseases (such as foot-and-mouth disease, vesicular stomatitis, and swine vesicular disease), significantly complicating the prevention and control of swine epidemics (1). SVV infection can also lead to sudden death in neonatal piglets, posing a serious threat to the healthy development of the pig industry and resulting in substantial economic losses (2). Originally identified as an adventitious agent in the cell culture, early isolates of SVV demonstrated no apparent virulence in natural hosts yet attracted significant research interest owing to their inherent oncolytic properties (3). A pivotal discovery occurred in 2007 when SVV was first characterized as the etiological agent of porcine idiopathic vesicular disease in Canada, marking its emergence as a swine pathogen. The virus was first detected in Guangdong Province, China, in 2015 (4), followed by progressive northward dissemination that ultimately resulted in widespread outbreaks throughout multiple provinces from 2017 onward (5).

SVV, the sole member of the *Senecavirus* genus within the *Picornaviridae* family, is a non-enveloped virus. Its genome comprises a single-stranded, non-segmented, positive-sense RNA approximately 7.3 kb in length, featuring both a 3′ polyadenylated tail and the lack of a 5′ cap structure (6, 7). The SVV genome contains 5′ and 3′ untranslated regions and a single open reading frame (ORF). The ORF encodes a polyprotein precursor that undergoes proteolytic processing according to the canonical picornaviral “L-P1-P2-P3” organization, ultimately yielding 12 mature proteins: four structural proteins (VP1–VP4) and seven non-structural proteins (2A, 2B, 2C, 3A, 3B [VPg], 3C protease, and 3D RNA-dependent RNA polymerase) (8).

The SVV 3C protease (3C^pro^) counteracts host innate immune responses through cleaving and degrading crucial molecules such as mitochondrial antiviral signaling protein (MAVS) (9), selective autophagy receptors SQSTM1/p62 and OPTN (optineurin) (10, 11), signal transducer and activator of transcription STAT2 (12), gasdermin D (GSDMD) (13), and nuclear factor kappa-B (NF-κB)-p65 (14), histone deacetylase 4 (HDAC4) (15), tripartite motif containing 32 (TRIM32) (16), and mRNA decapping enzyme 1A (DCP1A) (17). Additional research is required to examine the function of 3C^pro^ in other pathways, to comprehend its role in SVV infection, and to assess its potential as a target for therapeutic strategies.

The nuclear factor erythroid 2-related factor 2 (Nrf2) is a key regulator of antioxidation (18). Nrf2 function is restricted by kelch-like ECH-associated protein 1 (Keap1), which enhances Nrf2 ubiquitination and proteasomal degradation and inhibits its translocation into the nucleus, thereby maintaining homeostasis (19). Under oxidative stress, Nrf2 is released from Keap1 and rapidly translocated into the nucleus, followed by binding to antioxidant response elements (ARE) and activating the transcription of antioxidant genes, including heme oxygenase-1 (HO-1) and NAD(P)H quinone oxidoreductase 1 (NQO1) (20). The selective autophagy receptor p62/SQSTM1 activates Nrf2 by inactivating Keap1 (21, 22). The gene family of heme oxygenase (either HMOX or HO) encodes three distinct isoforms: HO-1, HO-2, and HO-3. Predominantly, HO-1 expression occurs in organs such as the liver, spleen, bone marrow, and the gastrointestinal tract. The rapid induction of HO-1 functions to counteract oxidative stress, inflammation, and other harmful stimuli (23–25). HO-1, an enzyme that can be induced by stress, has drawn significant interest. It serves as a crucial endogenous protective element capable of alleviating diverse cellular stresses. These include oxidative stress, endotoxins, hypoxia, heavy metals, heat shock, and inflammatory cytokines (26–28).

Oxidative stress is frequently triggered by viral infection. Recent research findings have indicated that Nrf2 and HO-1 are of great significance in antiviral function (29). For instance, herpes simplex virus 1 (HSV-1) and influenza A virus induce ferroptosis via the Nrf2-Keap1 pathway, contributing to viral encephalitis and nasal mucosal epithelial inflammation, respectively (30, 31). The dengue virus NS2B3 protein and SARS-CoV-2 nsp14 inhibit Nrf2/HO-1 expression to promote viral replication (32, 33). SARS-CoV-2 ORF3a degrades Nrf2, reducing cellular resistance to oxidative stress (34). An elevation in HO-1 expression hindered the replication of human respiratory syncytial virus (35), human immunodeficiency virus (HIV) (36), porcine circovirus type 3 (PCV3) (37), and porcine reproductive and respiratory syndrome virus (PRRSV) (38). PRRSV nsp5 inhibits the activation of the Nrf2/HO-1 pathway by targeting SQSTM1/p62 to antagonize its antiviral activity. However, the ways in which Nrf2/HO-1-related signals defend against SVV infection, as well as how SVV impacts the antioxidant response, are not fully comprehended.

In this study, we proved that SVV infection induced Nrf2/HO-1 degradation, thereby facilitating viral replication. In addition, SVV infection triggered oxidative stress within cells, resulting in elevated levels of reactive oxygen species (ROS) and malondialdehyde (MDA) while simultaneously causing a reduction in the levels of glutathione (GSH). Mechanistically, SVV 3C^pro^ induced the degradation of the Nrf2/HO-1 via the caspase pathway, which dramatically attenuates its antiviral effects. In summary, our results uncover the various tactics that SVV employs to elude the host’s antiviral reaction and offer a theoretical foundation for formulating novel antiviral approaches.

## RESULTS

### SVV infection induces Nrf2/HO-1 degradation and oxidative stress

**Fig 1:**
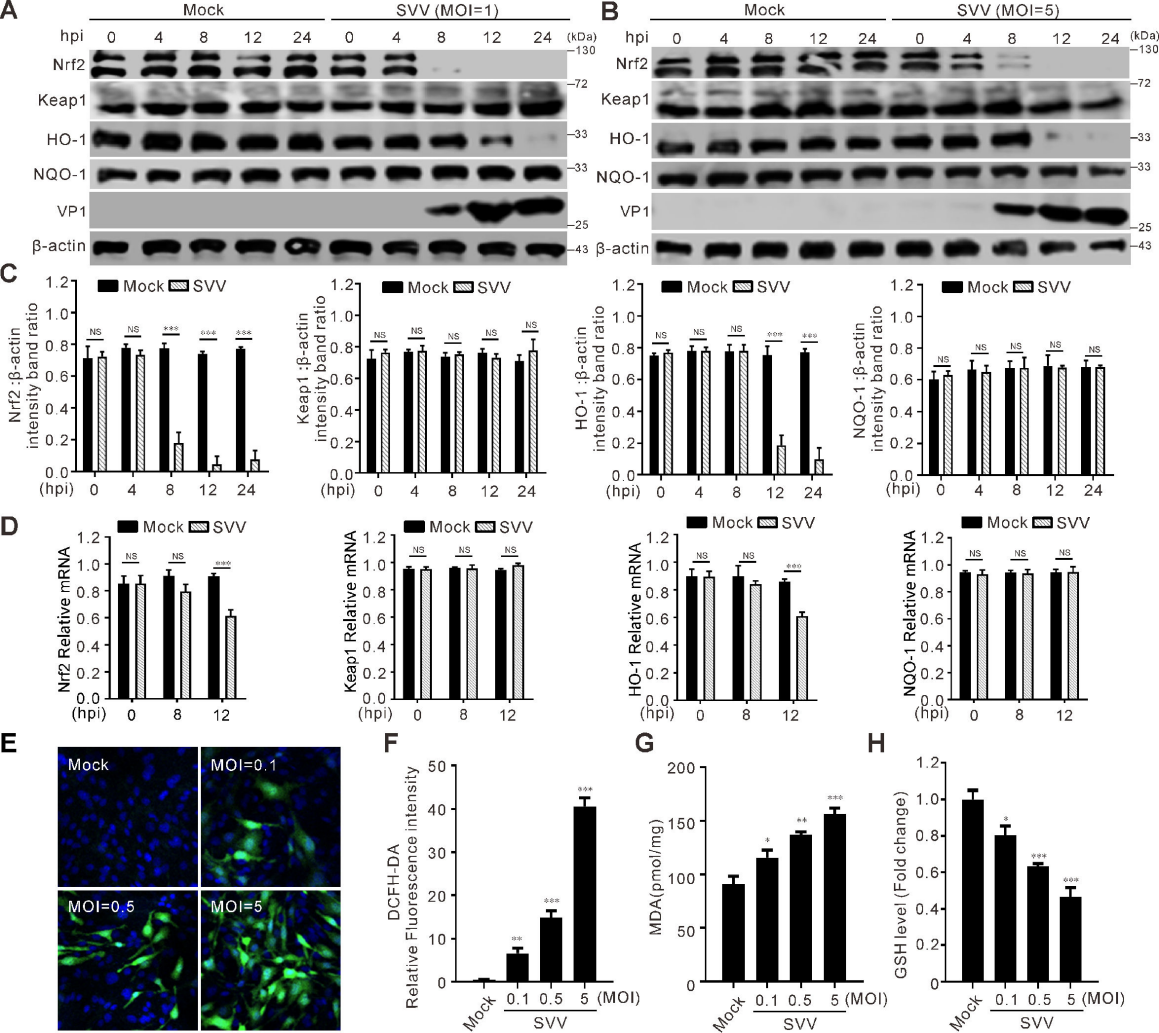
SVV infection inhibits Nrf2-HO-1 pathway. (**A and B**) BHK-21 cells were infected with SVV (multiplicity of infection [MOI] = 1 and 5). The cell lysates were collected at 0, 4, 8, 12, and 24 h post-infection (hpi) and analyzed by immunoblotting with antibodies against Nrf2, Keap1, HO-1, NQO-1, VP1, and β-actin as an internal control. (**C**) Quantification analysis of Nrf2, Keap1, HO-1, and NQO-1 protein expression levels from (**A**) using ImageJ. Error bars indicate mean ± SD from three independent infection experiments (***, *P* < 0.0010). (**D**) The transcriptional expression levels of Nrf2, Keap1, HO-1, and NQO-1 were analyzed using quantitative RT-PCR and normalized to β-actin mRNA. Error bars indicate mean ± SD from three independent infection experiments (MOI = 1) (***, *P* < 0.001). (**E**) BHK-21 cells were inoculated with SVV (MOI = 0.1, 0.5, and 5.0) and incubated for 12 h. Reactive oxygen species production was evaluated through the utilization of the fluorescent indicator DCFH-DA. A fluorescence microscope was used to take the images. (**F**) The fluorescence intensity ratio was analyzed by ImageJ. (**G and H**) BHK-21 cells were infected with SVV (MOI = 0.1, 1.0, and 5.0). The MDA and GSH levels were measured using an MDA assay kit and a GSH quantification kit, respectively. NS, not significant.

As a transcription factor involved in the regulation of numerous redox and antioxidant proteins, Nrf2 plays a key role in managing oxidative stress. HO-1 is a stress-activated enzyme that counteracts diverse cellular stresses. Initially, we determined whether SVV infection has an impact on the expression and transcription levels of Nrf2/HO-1 pathway. Infection of SVV with different dosages decreased the abundance of Nrf2 and HO-1 in a time-dependent manner (Fig. 1A through C). The mRNA transcriptional levels of Nrf2 and HO-1 were also reduced remarkably (Fig. 1D), which agreed with the protein level (Fig. 1A through C). These results suggested that SVV infection inhibits the Nrf2/HO-1 pathway. Subsequently, we investigated the influence of SVV infection on cellular oxidative stress. The fluorescent probe DCFH-DA was used to measure intracellular ROS levels. It was observed that as the doses of infection increased, there was a corresponding dose-dependent increase in the intensity of cellular fluorescence (Fig. 1E and F). Moreover, MDA, acting as an indicator of oxidative stress occurrence, exhibited a dose-dependent increase following SVV infection (Fig. 1G). SVV infection also caused a decrease in the levels of the antioxidant enzyme GSH (Fig. 1H). These results indicate that SVV infection triggers Nrf2/HO-1 degradation and oxidative stress.

### SVV infection inhibits Nrf2 translocation

**Fig 2:**
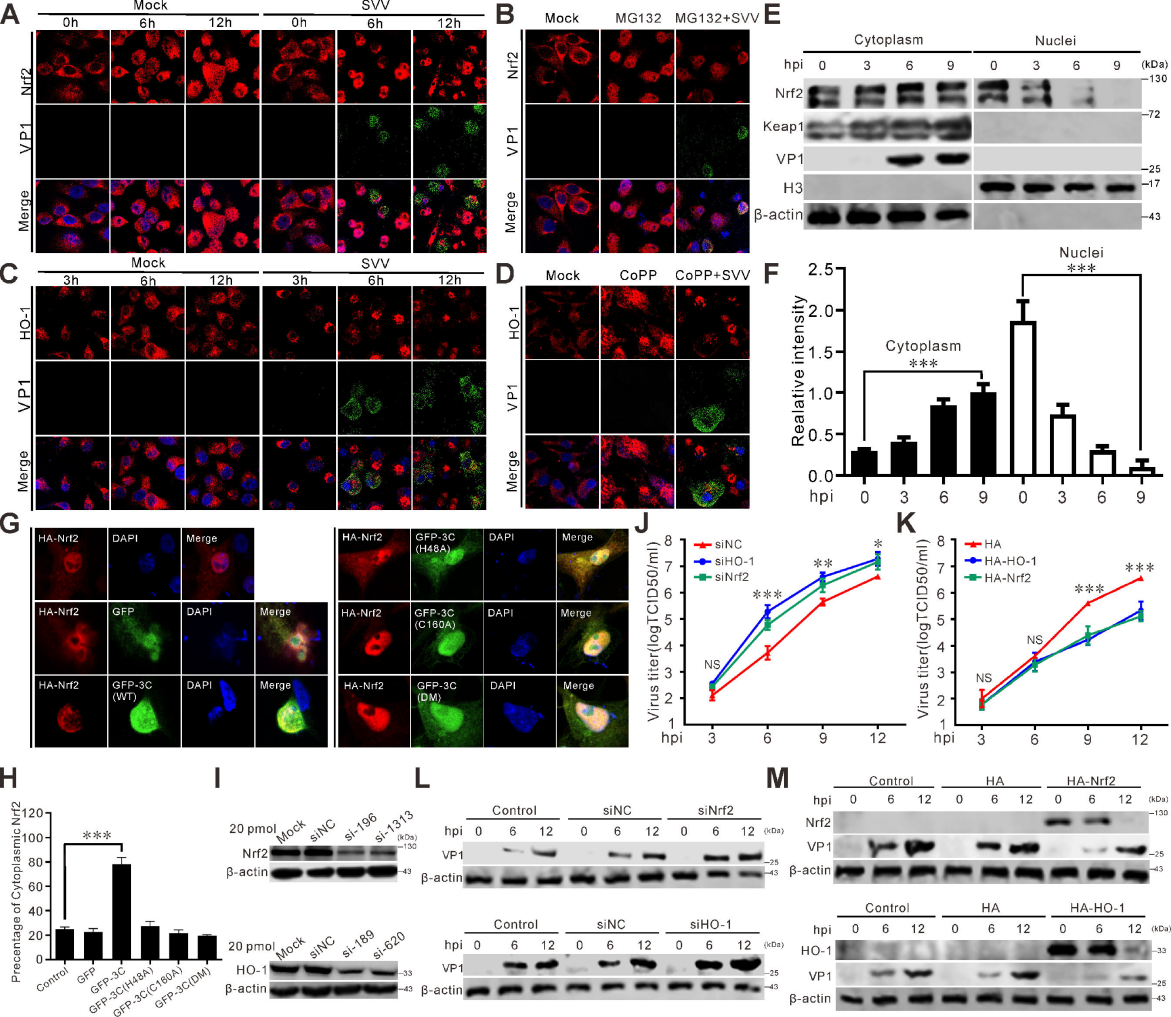
SVV infection retains Nrf2 in the cytoplasm. (**A and C**) BHK-21 cells infected with SVV (MOI = 0.5) or mock-infected with phosphate-buffered saline (PBS) with MG132 treatment (10 µM) (**A**) were monitored with confocal microscopy to examine subcellular localization of Nrf2 and HO-1 at 3, 6, and 12 hpi, respectively. Cells were stained with antibodies for detecting endogenous Nrf2 and HO-1 proteins (red), VP1 monoclonal antibody (green), and DAPI (blue), then examined by confocal microscopy. (**B**) BHK-21 cells mock-infected with PBS or infected with SVV (MOI = 0.5) with MG132 treatment (10 µM). Cells were stained with Nrf2 antibody (red), VP1 monoclonal antibody (green), and DAPI (blue), then examined by confocal microscopy. (**D**) BHK-21 cells mock-treated with dimethyl sulfoxide or CoPP or infected SVV (MOI = 0.5) with CoPP treatment (50 µM). Cells were stained with HO-1 antibody (red), VP1 monoclonal antibody (green), and DAPI (blue), then examined by confocal microscopy. (**E**) Nuclear and cytoplasmic fractions were collected at 0, 3, 6, and 9 hpi (MOI = 0.5) and analyzed by immunoblotting with antibodies against Nrf2, Keap1, histone H3, VP1, and β-actin as an internal control. (**F**) The relative gray intensity of cytoplasmic Nrf2 and nuclear Nrf2 was normalized against β-actin and histone H3, respectively, using ImageJ. Error bars indicate mean ± SD from three independent infection experiments (***, *P* < 0.001). (**G**) BHK-21 cells were transfected with HA-Nrf2 or co-transfected with HA-Nrf2 and either GFP, GFP-3C, GFP-3C-H48A, GFP-3C-C160A, or GFP-3C-DM (H48A-C160A). At 24 hpi, samples were processed for immunofluorescence staining using an HA antibody to detect exogenous HA-tagged Nrf2 proteins, followed by observation via confocal microscopy. (**H**) The graph shows the quantitative analysis of percentage of cytoplasmic Nrf2 from the results in panel **G**. Error bars indicate mean ± SD from three independent infection experiments (***, *P* < 0.001). (**I**) Immunoblot was used to analyze the expression of Nrf2 and HO-1 after siRNA transfection-mediated knockdown with antibodies against Nrf2 and HO-1, respectively, with β-actin as an internal control. (**J and K**) Growth curves of SVV (MOI = 0.5) after transfection with siRNAs, HA-Nrf2, and HA-HO-1 plasmids in BHK-21 cells. At 3, 6, 9, and 12 hpi, the total viruses were titrated with the TCID_50_ assay. Error bars indicate mean ± SD from three independent infection experiments. (*, *P* < 0.05; **, *P* < 0.01; ***, *P* < 0.001). (**L and M**) Immunoblot was used to analyze VP1 protein production in siRNAs, HA-Nrf2, and HA-HO-1 transfected BHK-21 cells at 6 and 12 hpi (MOI = 0.5).

To investigate the response of Nrf2 to SVV infection, we examined its subcellular localization using confocal microscopy. Fluorescence intensities of Nrf2 and HO-1 significantly decreased at the later stage of SVV infection (Fig. 2A and C). Treatment with MG132 facilitated Nrf2’s translocation into the nucleus, while infection with SVV led to a decrease in Nrf2’s nuclear localization (Fig. 2B). HO-1 agonist Cobalt protoporphyrin IX (CoPP) strongly induced HO-1 expression, but SVV infection reduced cytoplasmic HO-1 fluorescence intensity (Fig. 2D). Analysis of cytoplasmic and nuclear fractions confirmed that SVV infection retained Nrf2 in the cytoplasm (Fig. 2E and F). The cytoplasmic retention ratio of Nrf2 increased significantly following SVV infection, while nuclear fractions decreased (Fig. 2E and F). In the presence of green fluorescent protein (GFP)-3C, Nrf2 underwent relocalization from the nucleus and subsequent redistribution into the cytoplasm (Fig. 2G and H). In contrast, GFP-3C mutants deficient in protease activity had no impact on the subcellular distribution of Nrf2 (Fig. 2G and H). These findings indicate that SVV 3C^pro^ induces Nrf2 translocation. Transfection of BHK-21 cells with small interfering RNA (siRNA) or plasmids encoding hemagglutinin (HA)-tagged Nrf2 or HO-1 showed that siRNAs targeting Nrf2 and HO-1 efficiently inhibited their expression (Fig. 2I). Nrf2 and HO-1 knockdown increased viral titers and SVV replication (Fig. 2J and L), whereas Nrf2 and HO-1 overexpression reduced viral titers and VP1 production (Fig. 2K and M). These results demonstrate that SVV infection retains Nrf2 in the cytoplasm and that the Nrf2/HO-1 axis interferes with SVV infection.

### SVV 3C^pro^ targeted the Nrf2/HO-1 axis for degradation

**Fig 3:**
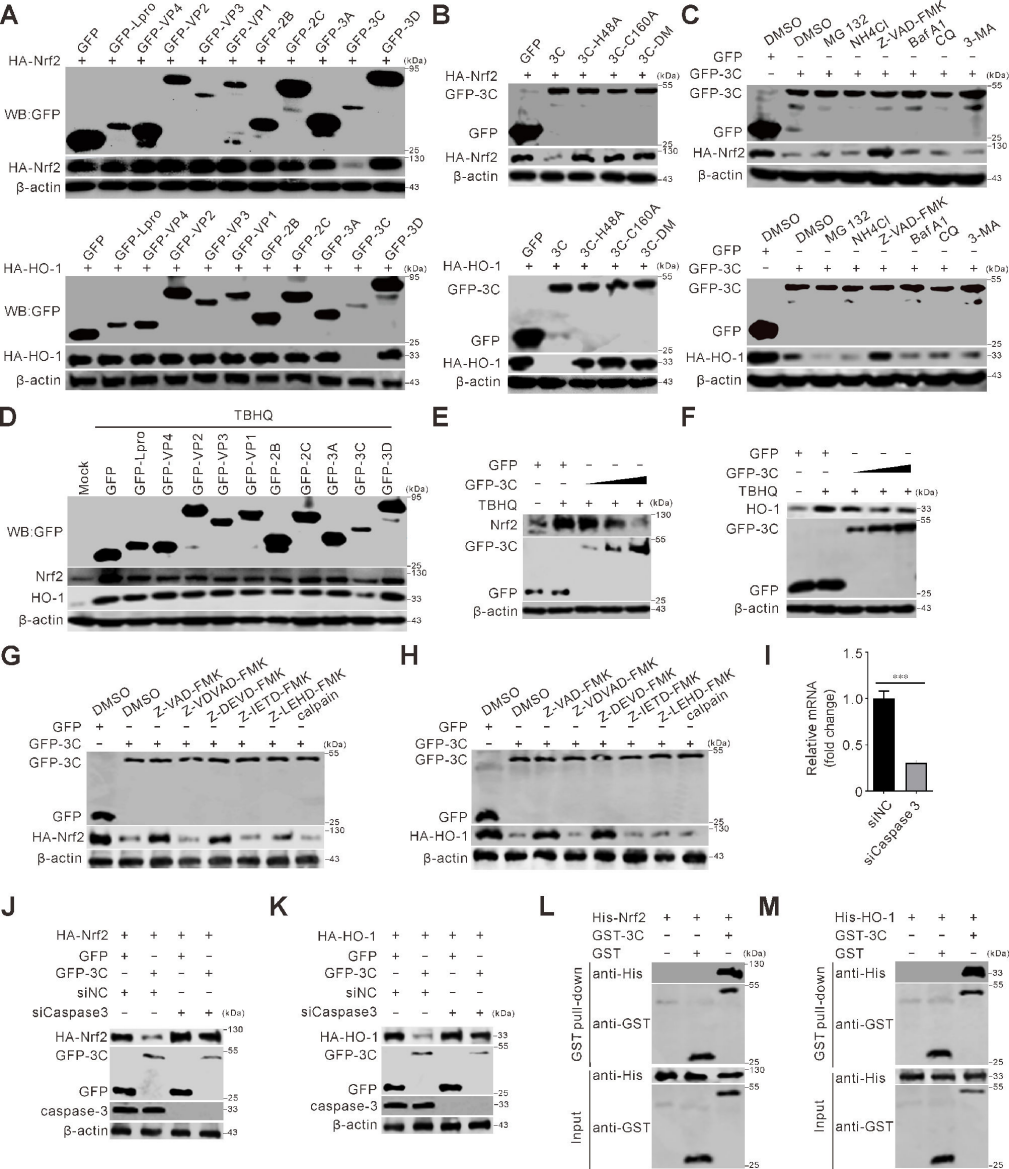
SVV 3C^pro^ targets Nrf2/HO-1 for degradation. (**A**) BHK-21 cells were co-transfected with influenza hemagglutinin (HA)-tagged Nrf2 and HO-1 with different green fluorescent protein (GFP)-tagged SVV protein expression plasmids for 24 h, respectively. Cell samples were subjected to immunoblotting with antibodies against HA, GFP, and β-actin. (**B**) BHK-21 cells grown on six-well plates were co-transfected with single mutant or double mutants GFP-tagged 3C^pro^, including 3C-H48A, 3C-C160A, and 3C-DM (H48A-C160A) with HA-tagged Nrf2, and HO-1 plasmids for 24 h, respectively. Cell samples were subjected to immunoblotting with antibodies against HA, GFP, and β-actin. (**C**) BHK-21 cells grown on six-well plates were co-transfected with GFP-3C and HA-tagged Nrf2 and HO-1 plasmids for 24 h, respectively, and GFP empty vector with HA as a control. MG132 (10 µM), NH_4_Cl (10 mM), Z-VAD-FMK (50 µM), Baf A1 (200 nM), CQ (40 µM), and 3-MA (25 mM) were added to cells at 12 h post-transfection (hpt) with DMSO as a control. Cells were collected at 24 hpt and subjected to immunoblot with antibodies against HA, GFP, and β-actin. (**D**) BHK-21 cells grown on six-well plates were co-transfected with GFP-tagged SVV protein expression plasmids for 24 h. TBHQ (10 µM) was added to the cells at 12 hpt. Cells were collected at 24 hpt and subjected to immunoblot with antibodies against Nrf2, HO-1, GFP, and β-actin. (**E and F**) BHK-21 cells grown on six-well plates were transfected with GFP-3C or GFP empty vector for 24 h. TBHQ (10 µM) was added to the cells at 12 hpt. Cells were collected at 24 hpt and subjected to immunoblot with antibodies against Nrf2 (**E**), HO-1 (**F**), GFP, and β-actin, respectively. (**G and H**) BHK-21 cells grown on six-well plates were co-transfected GFP-3C with HA-tagged Nrf2 and HO-1 plasmids for 24 h, respectively, and GFP empty vector as a control. Z-VAD-FMK (50 µM), Z-VDVAD-FMK (50 µM), Z-DEVD-FMK (50 µM), Z-IETD-FMK (50 µM), and Z-LEHD-FMK (50 µM) were added to cells at 12 hpt DMSO as a control. Cells were collected and subjected to immunoblot with antibodies against HA, GFP, and β-actin. (**I**) Quantitative RT-PCR (qRT-PCR) was performed to analyze the silencing efficiency of caspase-3. BHK-21 cells were transfected with caspase-3-targeting siRNA at a concentration of 20 pmol, while cells transfected with siNC served as negative controls. The graph shows the relative mRNA change from the results in panel I. Error bars indicate mean ± SD from three independent infection experiments (***, *P* < 0.001). (**J and K**) BHK-21 cells grown on six-well plates were co-transfected with GFP-3C, HA-Nrf2, or HA-HO-1 with siRNA targeting caspase-3. GFP empty vector and siNC were used as controls. Cells were collected at 36 hpt and subjected to immunoblot with indicated antibodies. (**L and M**) Purified GST-tagged 3C (GST-3C) and His-tagged Nrf2 or His-tagged HO-1 were incubated together and treated with Z-VAD-FMK (50 µM). The GST protein was used as a negative control. After incubation, the complexes were pulled down with glutathione-Sepharose beads and analyzed by Western blotting with anti-His and anti-GST antibodies.

To identify the viral protein responsible for antagonizing the Nrf2/HO-1 signaling pathway, SVV protein-expressing plasmids were co-transfected with HA-tagged Nrf2 and HO-1. The results indicated that SVV 3C^pro^ induced the degradation of Nrf2 and HO-1 *in vitro* (Fig. 3A). SVV 3C^pro^ plasmids with protease activity mutations (GFP-3C-H48A, GFP-3C-C160A, and GFP-3C-DM) showed that 3C^pro^-mediated degradation was protease activity dependent (Fig. 3B). Z-VAD-FMK treatment significantly reduced Nrf2 and HO-1 degradation. In contrast, inhibitors of the proteasome (MG132), lysosome (NH_4_Cl), and autophagy (bafilomycin A1, chloroquine [CQ], and 3-methyladenine [3-MA]) did not affect degradation (Fig. 3C), indicating that 3C^pro^ mediates degradation via the caspase pathway. The endogenous degradation of Nrf2 and HO-1 was examined in the presence of inducers. SVV protein-expressing plasmids transfected into BHK-21 cells and treated with tertiary butylhydroquinone (TBHQ) showed that SVV 3C^pro^ induced endogenous Nrf2 and HO-1 degradation (Fig. 3D). Dose-dependent reductions in Nrf2 and HO-1 protein levels were observed (Fig. 3E and F). These findings suggest that SVV 3C^pro^ degrades Nrf2 and HO-1 in a caspase-dependent manner. Subsequently, a set of caspase inhibitors—namely, the caspase-2 inhibitor Z-VDVAD-FMK, caspase-3 inhibitor Z-DEVD-FMK, caspase-8 inhibitor Z-IETD-FMK, and caspase-9 inhibitor Z-LEHD-FMK—was utilized to assess the role of caspases in 3C-induced reduction of Nrf2 and HO-1. As depicted in Fig. 3G and H, the caspase-3 inhibitor Z-DEVD-FMK restored the expression levels of Nrf2 and HO-1 under conditions where SVV 3C^pro^ was present. These observations indicate that the reduction of Nrf2 and HO-1 mediated by SVV 3C^pro^ relies on the activation of caspase-3. When caspase-3 was depleted via siRNA-mediated knockdown, the decrease in Nrf2 caused by SVV 3C^pro^ was alleviated. These results suggest that SVV 3C^pro^-mediated downregulation of Nrf2 is dependent on caspase-3 (Fig. 3J and K). In addition, His-tagged Nrf2 and HO-1 were purified, and their interactions with SVV 3C^pro^ were confirmed through GST pull-down assay *in vitro* (Fig. 3L and M). The results showed that His-tagged Nrf2 (His-Nrf2) and His-tagged HO-1 (His-HO-1) could specifically bind to GST-tagged 3C (GST-3C), but not to GST, indicating a direct interaction between 3C and Nrf2 as well as between 3C and HO-1.

### SVV infection-regulated ROS production is involved in inflammatory cytokines

**Fig 4:**
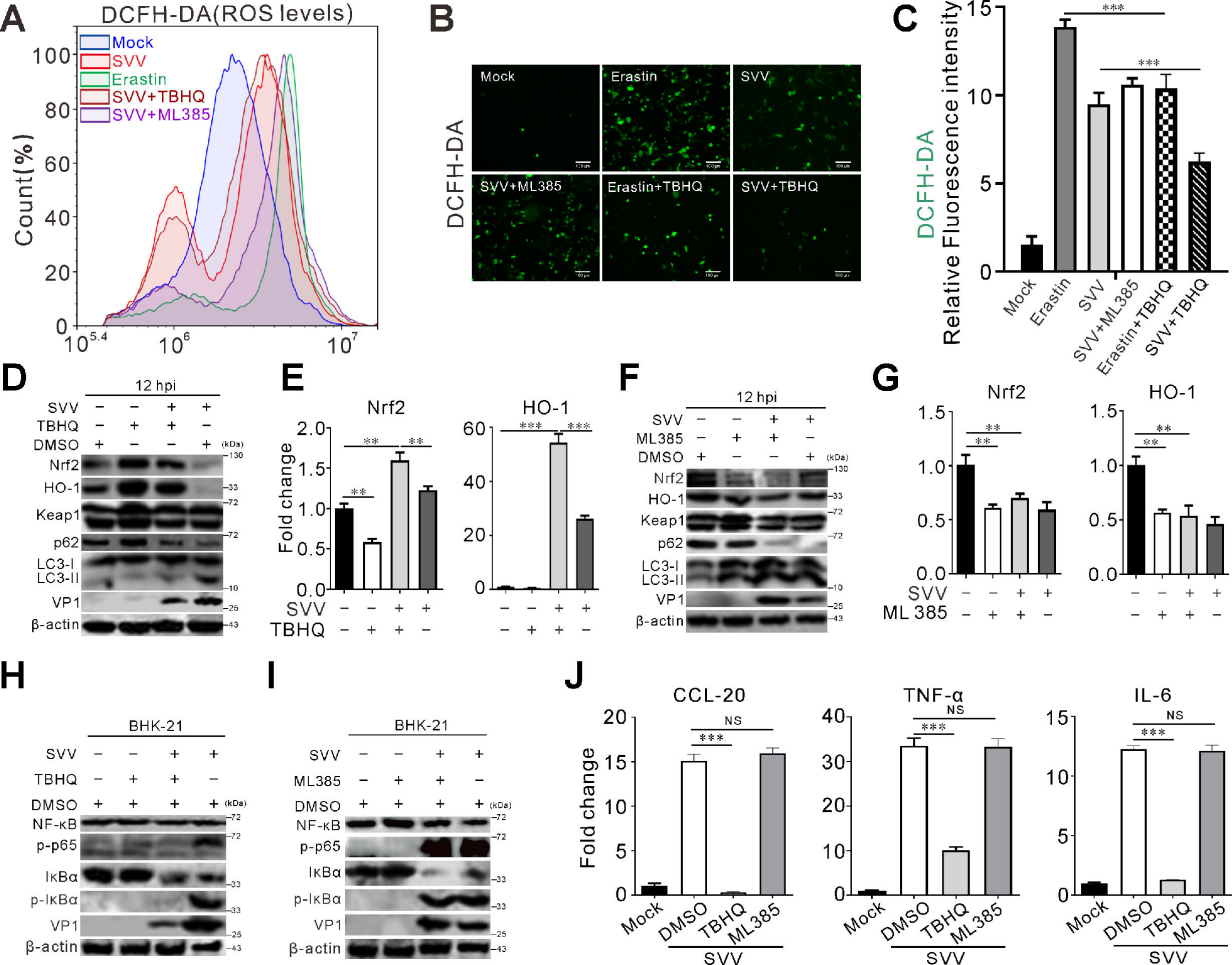
Nrf2 inhibits SVV replication. (**A and B**) BHK-21 cells were infected with SVV (MOI = 5) for 12 h, followed by treatment with the Nrf2 activator TBHQ (10 µM) or inhibitor ML385 (5 µM). At 12 hpt, intracellular ROS was stained by DCFH-DA probe (10 µM) and detected by flow cytometry and confocal microscopy, respectively, in indicated group. Erastin (10 µM) was included as a positive control for ROS induction. (**C**) The statistical results of the relative ROS fluorescence from (**B**) using ImageJ. Error bars indicate mean ± SD from three independent infection experiments (***, *P* < 0.001). (**D–G**) BHK-21 cells were infected with SVV (MOI = 5) for 12 h, then treated with either TBHQ (10 µM) (**D and E**) or ML385 (5 µM) (**F and G**). At 12 hpt, cells were collected and subjected to either Western blot analysis using antibodies against Nrf2, HO-1, p62, LC3, VP1, and β-actin (**D and F**) or to qRT-PCR to assess changes in Nrf2 and HO-1 mRNA expression levels (**E and G**). Error bars indicate mean ± SD from three independent infection experiments (**, *P* < 0.01; ***, *P* < 0.001). (**H–J**) BHK-21 cells were infected with SVV (MOI = 5) for 12 h, then treated with either TBHQ (10 µM) (**H and J**) or ML385 (5 µM) (**I and J**). At 12 hpt, cells were collected and subjected to either Western blot analysis using antibodies against NF-κB, p-p65, IκBα, p-IκBα, and β-actin (**H and I**) or qRT-PCR to determine the mRNA expression levels of CCL-20, TNF-α, and IL-6 (**J**). Error bars indicate mean ± SD from three independent infection experiments (***, *P* < 0.001).

Nrf2 is a critical regulator in influencing viral replication, inflammatory pathways, and ferroptosis (31, 32, 34, 39–42). The impact of Nrf2 on SVV replication was assessed using the Nrf2 agonist TBHQ and the Nrf2 inhibitor ML385. TBHQ treatment significantly reduced SVV-induced ROS production (Fig. 4A through C). Conversely, ML385 increased ROS accumulation (Fig. 4A through C), suggesting that Nrf2 is essential for controlling ROS generation. TBHQ also increased Nrf2 expression (Fig. 4D), whereas ML385 reduced Nrf2 levels (Fig. 4F). The autophagy-related receptors, including SQSTM1/p62 and the Nrf2/HO-1 axis, were upregulated with TBHQ treatment (Fig. 4D). TBHQ also inhibited SVV-induced LC3-II conversion and autophagy (Fig. 4D). In contrast, ML385 reduced Nrf2/HO-1 axis expression and induced autophagy (Fig. 4F). The mRNA levels of Nrf2 and HO-1 matched their protein expression (Fig. 4E and G). TBHQ, but not ML385 treatment, reduced inflammatory cytokine induction, mitigating the pro-inflammatory response to SVV (Fig. 4H through J). These findings suggest that SVV infection-modulated ROS generation is implicated in inflammatory cytokines.

### HO-1 agonist inhibits SVV infection

**Fig 5:**
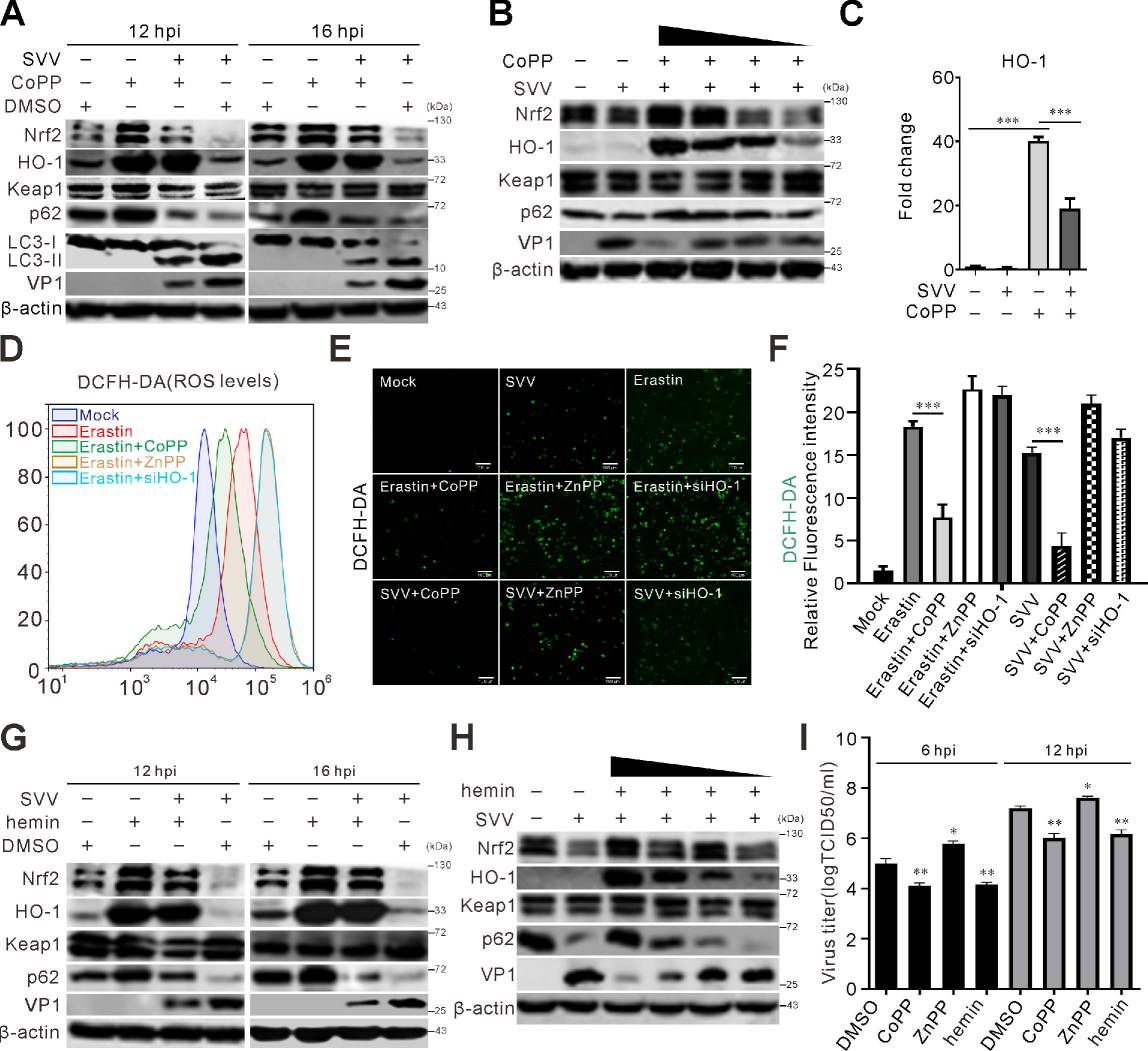
HO-1 agonist inhibits SVV infection. (**A**) BHK-21 cells were infected with SVV (MOI = 5) and treated with CoPP (50 µM). At 12 and 16 hpi, the cell lysates were collected and then analyzed by immunoblotting with indicated antibodies. (**B**) BHK-21 cells were infected with SVV (MOI = 5) and treated with CoPP at various concentrations (100, 50, 10, and 1 µM). At 12 hpi, immunoblot was used to analyze the protein expression with indicated antibodies. (**C**) BHK-21 cells were infected with SVV (MOI = 5) and treated with CoPP (50 µM). The transcriptional expression level of the indicated gene was analyzed using qRT-PCR and normalized to β-actin mRNA. Error bars indicate mean ± SD from three independent infection experiments (***, *P* < 0.001). (**D and E**) BHK-21 cells were treated with erastin or in the presence of HO-1 activator CoPP (50 µM) and inhibitor ZnPP (10 µM) or transfected with siHO-1. ROS was measured using DCFH-DA (10 µM) and examined by flow cytometry (**D**) and confocal microscopy (**E**), respectively. (**F**) Relative fluorescence intensity in BHK-21 cells from panel** E**. Error bars indicate mean ± SD from three independent infection experiments (***, *P* < 0.001). (**G**) BHK-21 cells were infected with SVV (MOI = 5) and treated with HO-1 inducer hemin (50 µM). The cell lysates were collected at 12 and 16 hpi and analyzed by immunoblotting with indicated antibodies. (**H**) BHK-21 cells were infected with SVV (MOI = 5) and treated with varying concentrations of hemin (100, 50, 10, and 1 µM). At 12 hpi, immunoblot was used to analyze the protein expression with indicated antibodies. (**I**) BHK-21 cells were infected with SVV (MOI = 0.5), followed by treatment with CoPP (50 µM), ZnPP (10 µM), or hemin (50 µM). At 6 and 12 hpi, the viral titers were determined with TCID_50_ assay. Error bars indicate mean ± SD from three independent infection experiments (*, *P* < 0.05; **, *P* < 0.01).

HO-1, a downstream product of Nrf2 involved in stress detoxification, plays a crucial role in regulating ferroptosis and type I interferon (IFN-I) production (43–45). Treatment with the HO-1 agonist CoPP extremely increased HO-1 protein and mRNA levels (Fig. 5A through C), enhanced Nrf2 and p62 accumulation (Fig. 5A through C). CoPP activated the Nrf2/HO-1 axis while inhibiting LC3-II conversion (Fig. 5A and B). CoPP-induced dose-dependent increases in Nrf2 and HO-1 were reversed by SVV infection (Fig. 5B). CoPP suppressed erastin-induced lipid ROS generation, whereas zinc protoporphyrin (ZnPP) (a HO-1 inhibitor) and HO-1 knockdown enhanced ROS accumulation (Fig. 5D through F). Hemin is capable of inducing the expression of HO-1. As a typical inducer of HO-1, hemin exhibits antiviral effects against a number of viruses, such as hepatitis A virus (36, 38, 46, 47). As expected, treatment with hemin significantly increased the HO-1 protein level in a dose-dependent manner (Fig. 5G and H). Hemin was shown to exhibit antiviral activity against SVV; hemin led to the reduction of viral capsid protein in a dose-dependent fashion (Fig. 5G and H). CoPP and hemin treatment showed greatly reduced viral titers, while ZnPP promotes SVV replication (Fig. 5I). These results indicate that SVV induces HO-1 degradation to facilitate viral replication.

### HO-1 metabolite suppresses SVV replication

**Fig 6:**
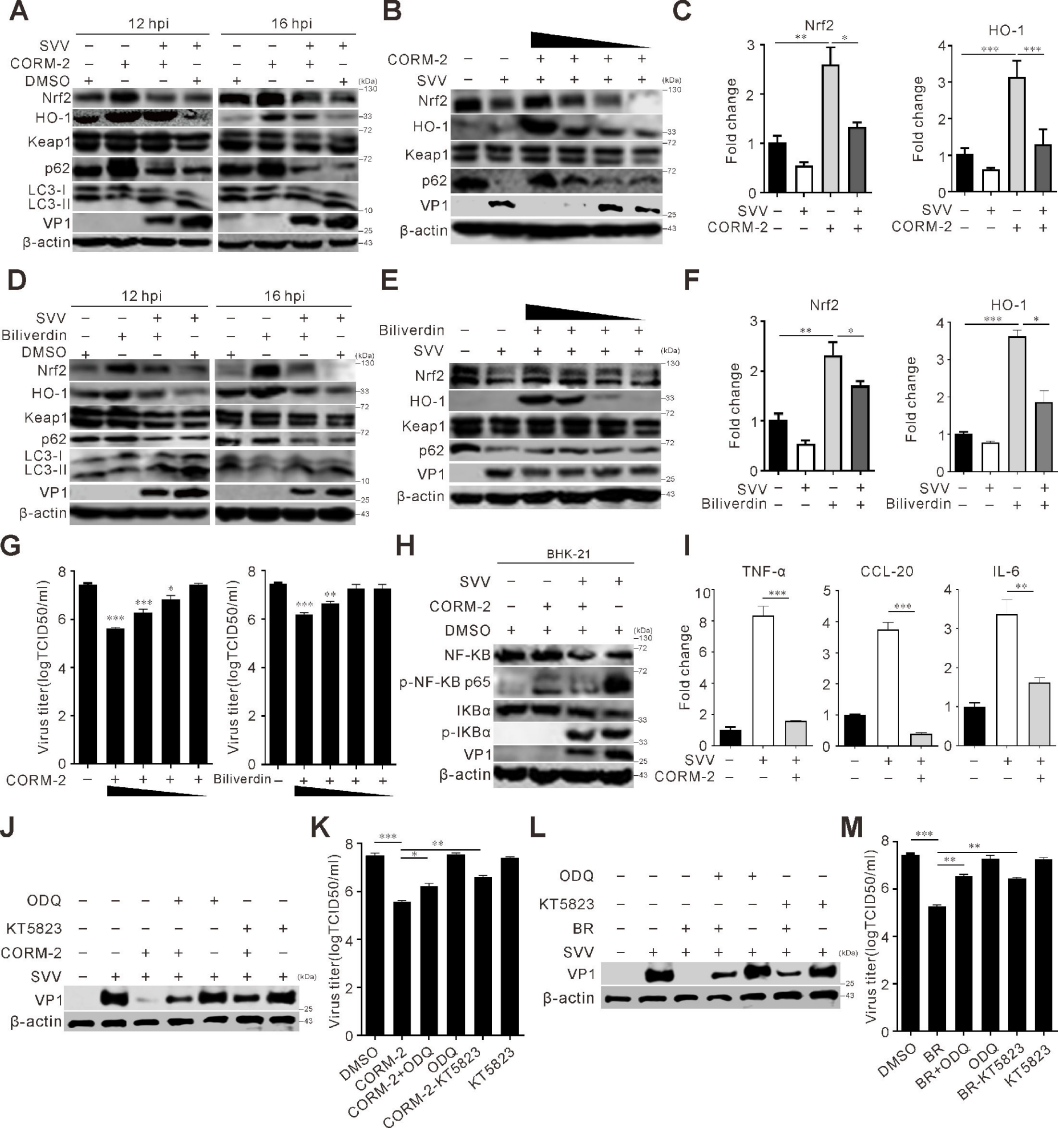
HO-1 metabolite inhibits SVV replication. (**A and D**) BHK-21 cells were infected with SVV (MOI = 5) and treated with CORM-2 (50 µM) (**A**) and biliverdin (50 µM) (**D**), respectively. At 12 and 16 hpi, the cell lysates were collected and analyzed by immunoblotting with indicated antibodies. (**B and E**) BHK-21 cells were infected with SVV (MOI = 5) and treated with CORM-2 and biliverdin with various concentrations (100, 50, 10, and 1 µM), respectively. At 12 hpi, immunoblot was used to analyze the protein expression with indicated antibodies. (**C and F**) BHK-21 cells were infected with SVV (MOI = 5) and treated with CORM-2 (50 µM) (**C**) and biliverdin (50 µM) (**F**), respectively. The transcriptional expression level of the indicated gene was analyzed using qRT-PCR and normalized to β-actin mRNA. Error bars indicate mean ± SD from three independent infection experiments (*, *P* < 0.05; ***, *P* < 0.001). (**G**) BHK-21 cells were infected with SVV (MOI = 0.5) and treated with CORM-2 and biliverdin at various concentrations (100, 50, 10, and 1 µM), respectively. The virus productions at 12 hpi were titrated by TCID_50_ assay. (**H and I**) BHK-21 cells were infected with SVV (MOI = 5) and treated with CORM-2 (50 µM). At 12 hpi, the cells were collected and subjected to immunoblot analysis of NF-κB pathway protein expression using the indicated antibodies (**H**) or qRT-PCR quantification of CCL-20, TNF-α, and IL-6 mRNA levels (**I**). Error bars indicate mean ± SD from three independent infection experiments (**, *P* < 0.01; ***, *P* < 0.001). (**J and K**) BHK-21 cells were either exposed to CORM-2 (50 µM), ODQ (10 µM), or KT5823 (1 µM) or left unexposed, followed by SVV infection. At 24 hpi, the VP1 protein and SVV titers were determined by Western blot (**J**), and TCID_50_ (**K**) was determined. (**L and M**) BHK-21 cells were treated with BR (50 µM), ODQ (10 µM), or KT5823 (1 µM) or kept untreated, and then infected with SVV. At 24 hpi, the VP1 protein and SVV titers were determined by Western blot (**L**) and TCID_50_ (**M**). Error bars indicate mean ± SD from three independent infection experiments. (*, *P* < 0.05; **, *P* < 0.01; ***, *P* < 0.001).

To investigate the mechanism of HO-1-mediated anti-SVV infection, we assessed the effects of the HO-1 enzymatic products carbon monoxide (CO) and biliverdin (BV) on SVV replication. Treatment with tricarbonyldichlorouthenium(II) dimer (CORM-2) (a CO donor) or BV led to a dose-dependent reduction in viral replication (Fig. 6A, B, D, E and, G). This suggests that BV and CO contribute to SVV inhibition. CORM-2 treatment increased the protein and mRNA levels of Nrf2 and HO-1 (Fig. 6A through C). Additionally, LC3-II expression and p62 degradation were inhibited, indicating reduced SVV-induced autophagy (Fig. 6A and B). BV treatment increased the protein and mRNA levels of Nrf2 (Fig. 6D–F). The inhibitory effect of SVV infection decreased with lower BV and CORM-2 concentrations (Fig. 6B and E). Consistent with these findings, CORM-2 inhibited SVV-induced NF-κB pathway activation and inflammatory factor transcription (Fig. 6H and I). A previous research has reported the involvement of the cyclic guanosine monophosphate (cGMP)/protein kinase G (PKG) pathway in CO-mediated antiviral effects (48). This finding prompted an investigation into the role of the cGMP/PKG signaling pathway in regulating SVV replication. Two specific inhibitors were employed: ODQ (1H-[1,2,4]oxadiazolo[4,3a]quinoxalin-1-one), which targets guanylate cyclase (soluble guanylyl cyclase [sGC]), and KT5823, which inhibits PKG. Co-treatment with ODQ and KT5823 partially abrogated the CORM-2-mediated reduction in VP1 protein levels and SVV titers (Fig. 6J and K). However, individual treatment with either ODQ or KT5823 failed to inhibit SVV replication (Fig. 6J and K), indicating that SVV replication in BHK-21 cells is dependent on the intact cGMP/PKG signaling pathway. These data thus support the conclusion that the anti-SVV activity of CO is dependent on CO-induced upregulation of HO-1 and subsequent activation of the cGMP/PKG signaling pathway. It is widely recognized that sGC gets activated and cGMP levels rise in cells generating nitric oxide (NO) (49). In this study, specific inhibitors (ODQ and KT5823) targeting NO-induced sGC or cGMP-dependent protein kinase (PKG) were employed to explore the connection between sGC, cGMP, and SVV replication. As shown in Fig. 6L and M, ODQ or KT5823 significantly reversed the NO-mediated inhibition of SVV replication in the presence of berberine (BR): this was evident from the increased expression of the VP1 protein and higher SVV titers. These results suggest that the cGMP/PKG signaling pathway is involved in suppressing SVV infection and plays a vital part in the NO-mediated anti-SVV activity.

### N-acetyl-L-cysteine blocks SVV replication via inhibiting autophagy

**Fig 7:**
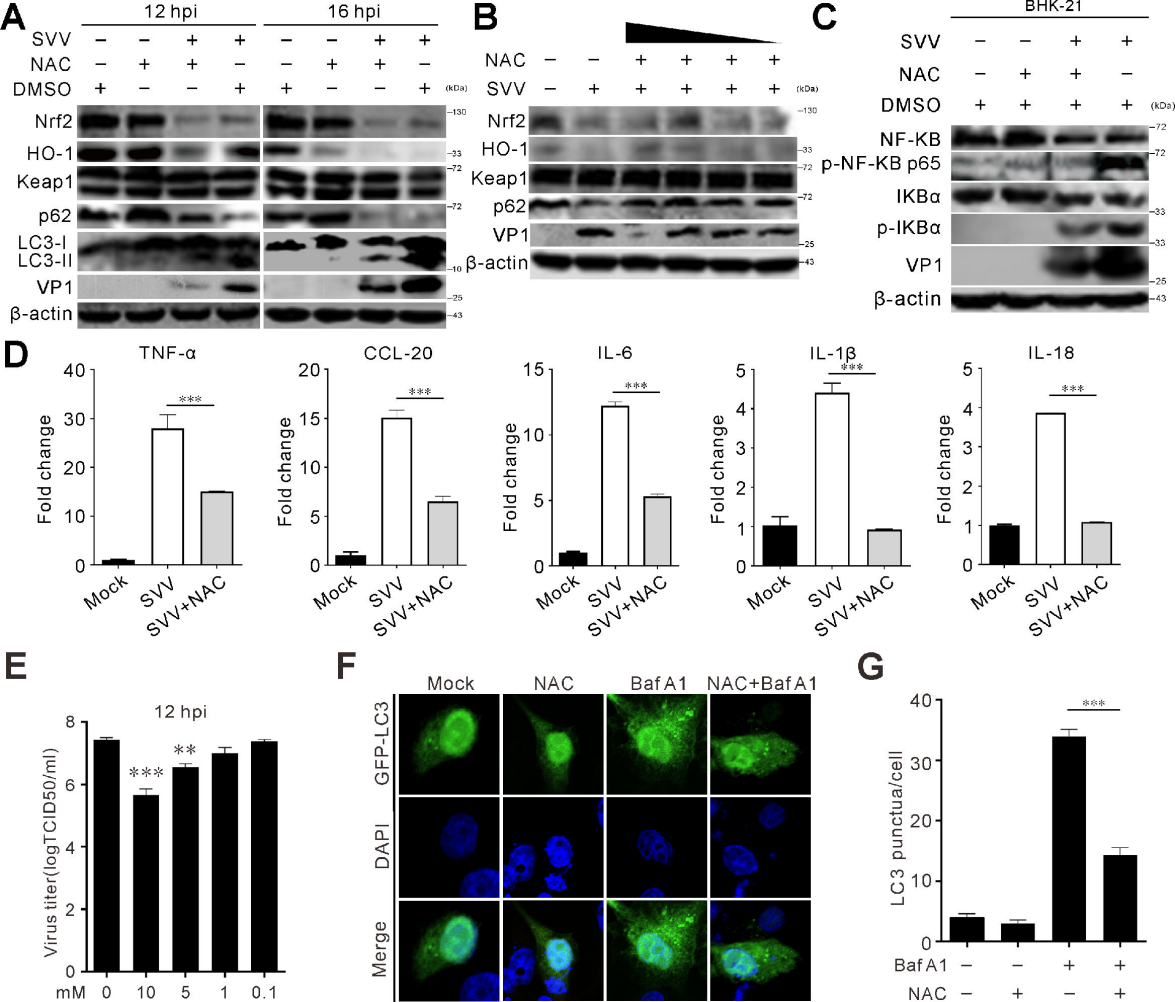
NAC blocks SVV replication via inhibiting autophagy. (**A**) BHK-21 cells were infected with SVV (MOI = 5) and treated with NAC (5 mM). At the indicated times, the cell lysates were collected and analyzed by immunoblotting with antibodies against Nrf2, HO-1, Keap1, p62, LC3, VP1, and β-actin. (**B**) BHK-21 cells were infected with SVV (MOI = 5) and treated with NAC at various concentrations (10.0, 5.0, 1.0, and 0.1 mM). At 12 hpi, immunoblot was used to analyze the protein expression with indicated antibodies. (**C and D**) BHK-21 cells were infected with SVV (MOI = 5) and treated with NAC (5 mM). At 12 hpi, the cells were collected and analyzed by immunoblotting for NF-κB signaling proteins (**C**) or qRT-PCR quantification for cytokine mRNAs (CCL-20, TNF-α, IL-6, IL-1β, and IL-18) (**D**). Error bars indicate mean ± SD from three independent infection experiments (***, *P* < 0.001). (**E**) BHK-21 cells were infected with SVV (MOI = 0.5) and treated with NAC at various concentrations (10.0, 5.0, 1.0, and 0.1 mM). At 12 hpi, the viral titers were determined with TCID_50_ assay. Error bars indicate mean ± SD from three independent infection experiments (**, *P* < 0.01; ***P < 0.001). (**F and G**) GFP-LC3 was transfected into BHK-21 cells, and treatment with NAC (5 mM) was conducted for 24 h. Fluorescent microscopy was employed to detect LC3 puncta (left), while the quantification of LC3 puncta per cell is displayed on the right. Error bars indicate mean ± SD from three independent infection experiments (***P < 0.001).

N-acetyl-L-cysteine (NAC) is a ROS scavenger with antioxidant and anti-inflammatory properties. NAC treatment inhibited SVV-induced LC3-II conversion and enhanced SQSTM1/p62 accumulation (Fig. 7A and B). Viral titers and VP1 production were reduced in a dose-dependent manner with NAC treatment (Fig. 7B and E). NAC treatment also attenuated SVV-induced NF-κB phosphorylation (Fig. 7C) and inhibited inflammatory factor transcription (TNF-α, CCL-20, IL-6, IL-1β, and IL-18) (Fig. 7D). Compared with control cells, a marked rise in LC3 puncta was detected in cells treated with Baf A1 (Fig. 7F and G). Notably, treatment with NAC eliminated the Baf A1-triggered formation of LC3 puncta (Fig. 7F and G). These results demonstrate that NAC suppresses SVV replication by inhibiting autophagy and inflammatory responses.

### HO-1 suppresses SVV replication via IFN-I pathway

**Fig 8:**
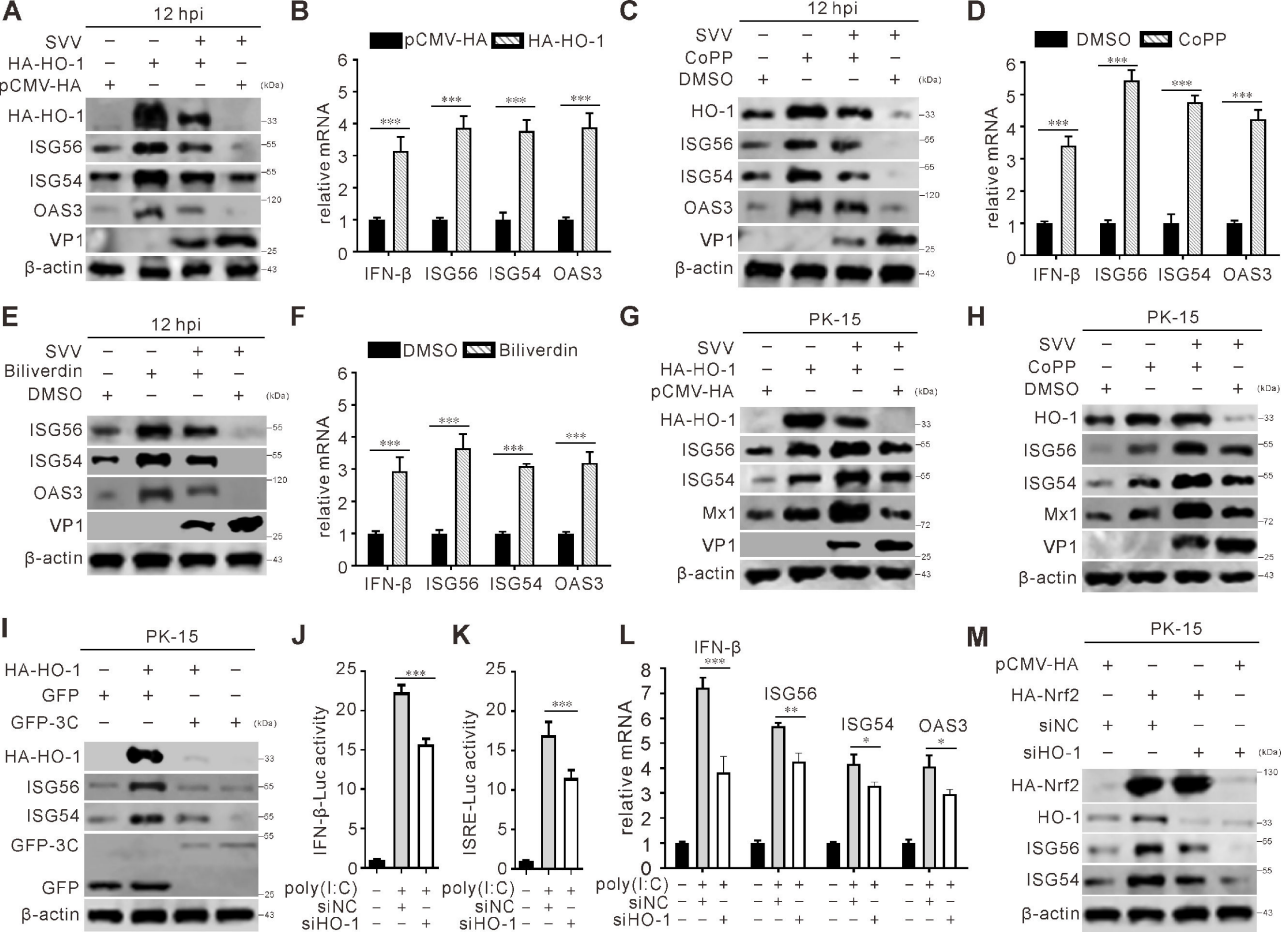
HO-1 inhibits SVV replication through the IFN-I pathway. (**A and G**) BHK-21 cells and PK-15 cells were transfected with HA-HO-1 and infected with SVV (MOI = 5), respectively. At 12 hpi, immunoblot was used to analyze the protein expression with indicated antibodies. (**B**) BHK-21 cells were transfected with HA-HO-1. The transcriptional expression level of the indicated gene was analyzed using qRT-PCR and normalized to β-actin mRNA. Error bars indicate mean ± SD from three independent infection experiments (***, *P* < 0.001). (**C and H**) BHK-21 cells and PK-15 cells were infected with SVV (MOI = 5) and treated with CoPP (50 µM), respectively. At 12 hpi, immunoblot was used to analyze the protein expression with indicated antibodies. (**D and F**) BHK-21 cells were infected with SVV (MOI = 5) and treated with CoPP (50 µM) and biliverdin (50 µM), respectively. The transcriptional expression level of the indicated gene was analyzed using qRT-PCR and normalized to β-actin mRNA. Error bars indicate mean ± SD from three independent infection experiments (***, *P* < 0.001). (**E**) BHK-21 cells were infected with SVV (MOI = 5) and treated with biliverdin (50 µM). At 12 hpi, immunoblot was used to analyze the protein expression with indicated antibodies. (**I**) PK-15 cells grown on six-well plates were co-transfected with GFP-3C and HA- HO-1 for 24 h, respectively, and GFP empty vector was used as a control. At 24 hpt, cells were collected and subjected to immunoblot with indicated antibodies. (**J and K**) HEK-293T cells plated in 24-well plates were transfected with 250 ng of IFN-β-Luc and ISRE-Luc separately, along with 25 ng of pRL-TK and either 250 ng of siHO-1 or siNC. After 24 h, HEK-293T cells were treated with poly(I:C) (2 ug/ml). Then the samples were prepared and analyzed by luciferase assays. (**L**) HEK-293T cells plated in 24-well plates were transfected with siHO-1 or siNC. After 24 h, the cells were treated with poly(I:C) (2 μg/mL). Then the samples were prepared and analyzed by qRT-PCR.Error bars indicate mean ± SD from three independent infection experiments. (*, *P* < 0.05; **, *P* < 0.01; ***, *P* < 0.001). (**M**) PK-15 cells grown on six-well plates were co-transfected with HA-Nrf2 and siRNA targeting HO-1; the GFP empty vector and siNC were used as controls. At 36 hpt, cells were collected and subjected to immunoblot with indicated antibodies.

**Fig 9:**
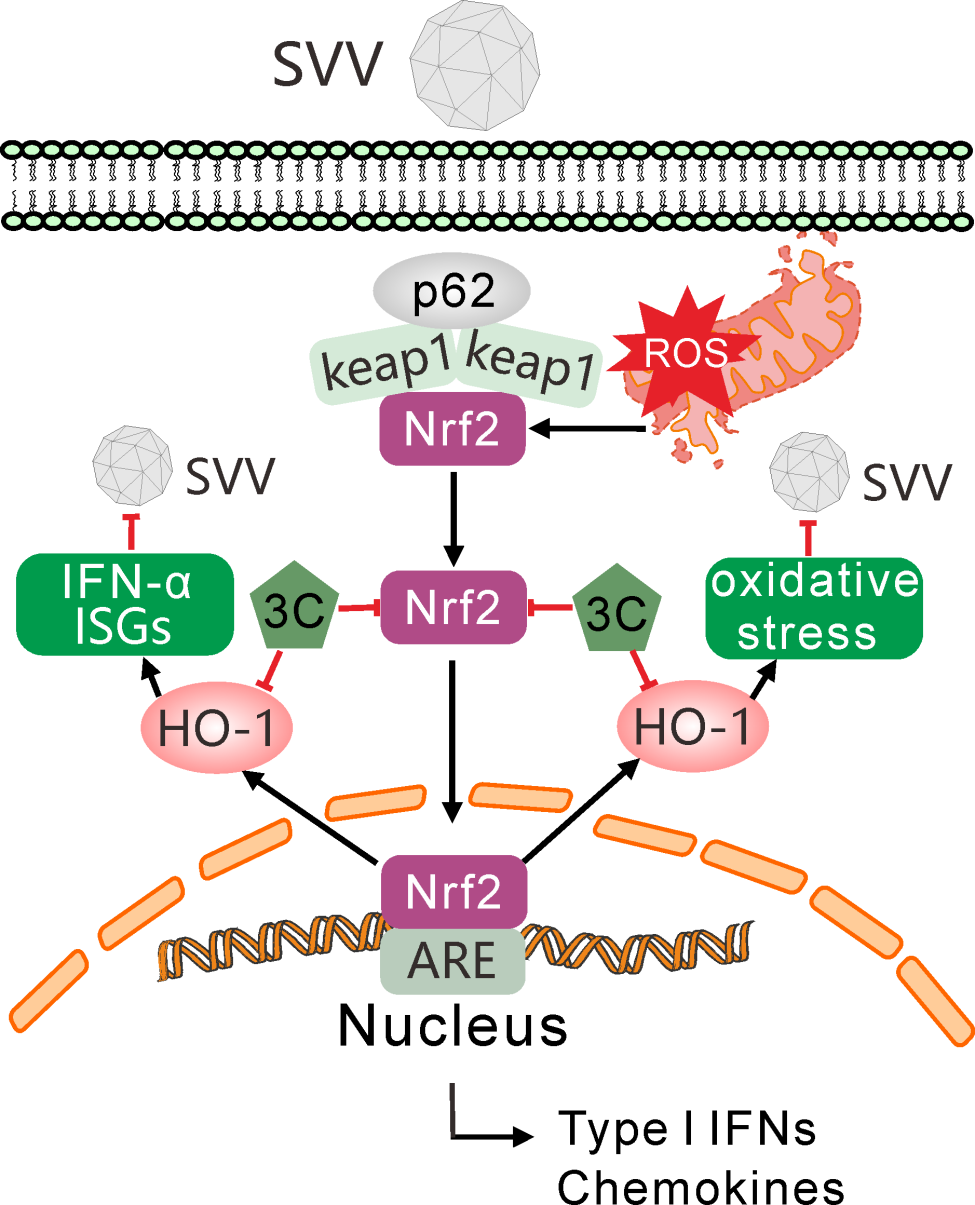
Proposed model for SVV 3C proteinase (3C^pro^) targeting Nrf2/HO-1 to facilitate viral replication. SVV infection elicited cellular oxidative stress through the induction of reactive oxygen species (ROS) production, glutathione depletion, and the suppression of the nuclear factor erythroid 2-related factor 2 (Nrf2)/heme oxygenase-1 (HO-1) pathway. Mechanistically, the viral 3C^pro^ targets the Nrf2/HO-1 axis for degradation via caspase pathway, thereby promoting viral replication. The overexpression of Nrf2/HO-1 exerted antiviral activity against SVV, whereas inhibition of this pathway significantly enhanced viral proliferation. HO-1 metabolic products carbon monoxide (CO) and biliverdin (BV) exhibited inhibition of SVV replication. Additionally, HO-1 promotes IFN-I response and ISG expressions, which contribute to its antiviral mechanism.

HO-1 was also shown to interact with interferon regulatory factor 3 (IRF-3), independent of its enzyme activity, which activated the type I IFN response to inhibit infection by influenza A virus (50). To determine whether HO-1 can modulate IFN-I signaling to contribute to the antiviral response, overexpression of HO-1 was initially used to evaluate the impact of HO-1 on the expression of interferon-stimulated genes (ISGs), such as ISG56, ISG54, and 2′-5′-oligoadenylate synthetase 3 (OAS3). The findings indicated that ectopic expression of HO-1 remarkably increased the levels of ISGs (Fig. 8A). Next, we investigated the effect of the HO-1 agonist CoPP and the HO-1 product biliverdin on the expressions of ISGs. The results indicated that CoPP and biliverdin enhanced the protein expressions of these ISGs (Fig. 8C and E), as well as the mRNA transcriptional levels of IFN-β and ISGs (Fig. 8B, D, and F). Consistent outcomes were observed in IFN-proficient porcine kidney-15 (PK-15) cells as well, where both HO-1 overexpression and treatment with the HO-1 agonist CoPP suppress SVV replication via ISGs (Fig. 8G and H). SVV 3C^pro^ was able to suppress ISGs by degrading HO-1 when overexpressed (Fig. 8I). In addition, dual luciferase reporter assay was employed to assess the impact of HO-1 on the promoter activity of IFN-I and ISRE. The results indicated that siRNA-mediated knockdown of HO-1 led to a partial suppression of the poly(I:C)-induced increases in IFN-β promoter activity and ISRE promoter activity (Fig. 8J and K). Suppression of HO-1 expression caused a significant attenuation of the levels of ISGs (Fig. 8L). Overexpression of Nrf2 led to increased levels of ISGs, but this effect was inhibited when HO-1 was knocked down via siRNA; these results demonstrated that Nrf2 regulates the IFN pathway in a HO-1-dependent manner (Fig. 8M). Taken together, these findings suggest that HO-1 promotes IFN-I response and ISG expressions, which contribute to its antiviral mechanism (Fig. 9).

## DISCUSSION

When faced with abnormal oxidative stress, cells possess diverse systems to safeguard against the damage and inflammation caused by oxidative stress. Nrf2 was recognized as a key controller of the cell’s resistance to oxidants. Plenty of studies have been reported about either the activation or the suppression of Nrf2 in cells by various viruses (51). Significantly, the generation of ROS and the ensuing stress response have been associated with the onset of inflammation and the progression to the more severe manifestations of the disease. As a main cytoprotective factor against ROS, the Nrf2/HO-1 axis is mainly expressed in the respiratory tract, most notably in lung tissue. It plays a crucial part in mediating anti-inflammatory activities and shielding tissue from the harm resulting from oxidative stress (52–54). In this study, we show that the SVV makes use of the 3C protease to target Nrf2/HO-1 for degradation.

ROS have crucial functions in programmed cell death (PCD), such as necroptosis, apoptosis, autophagy, and ferroptosis (55). The accumulation of excessive ROS attacks biomembranes and propagates lipid peroxidation chain reactions, thus inducing PCD (55). We examined ROS generation in BHK-21 cells after SVV infection and found that SVV infection or erastin treatment significantly induced ROS and lipid ROS accumulation (Fig. 1E and 1F). Further studies indicated a decline in GSH levels after SVV infection (Fig. 1H). Additionally, the MDA levels were upregulated following SVV infection (Fig. 1G). Enterovirus 71 induces neural cell death by increasing ROS production and attenuating the Nrf2/HO-1 pathway (56). As an inducible defense system in response to oxidative stress, the basal level of Nrf2/HO-1 is relatively low but can be significantly increased in response to diverse stimuli, such as hypoxia, ROS, and pathogenic infections (57–59). Nrf2 is a redox-responsive factor that protects cells from oxidative stress and injury, whereas the Nrf2/HO-1 pathway plays a key role in inflammation, antiviral response, and ferroptosis (32, 33, 60, 61). Infection with herpes simplex virus-1 and influenza A virus induces ferroptosis via the Nrf2-Keap1 pathway (30, 31). SARS-CoV-2 infection led to a significant reduction in both HO-1 mRNA levels and protein levels (34). Our results showed that the Nrf2/HO-1 pathway was inhibited by SVV infection (Fig. 1A through D).

The activation of Nrf2 can be utilized to counteract SARS-CoV-2, offering strong cytoprotection through the restoration of redox and protein homeostasis, the promotion of inflammation resolution, and the facilitation of repair (62). The Nrf2 agonists, 4-octyl-itaconate (4-OI) and dimethyl fumarate (DMF), trigger a cellular antiviral process that strongly suppresses the replication of SARS-CoV-2 via an IFN-I-reliant mechanism (41). SARS-CoV-2 NSP14 undermines the activation of Nrf2/HO-1 through targeting Sirtuin 1, whereas the metabolite BV generated by HO-1 can effectively suppress SARS-CoV-2 replication (33). Our findings demonstrate that Nrf2 activation, either through TBHQ treatment or genetic overexpression, exerts potent antiviral effects against SVV by significantly suppressing viral replication while simultaneously attenuating key pathogenic processes including ROS accumulation, NF-κB pathway activation, and pro-inflammatory cytokine production (Fig. 2K, M, 4A, B, D, H and, J). Conversely, pharmacological blockade of Nrf2 using ML385 or genetic silencing markedly enhanced SVV replication efficiency and exacerbated viral pathogenesis, as evidenced by elevated oxidative stress, heightened NF-κB signaling, and amplified inflammatory responses (Fig. 2J, L, 4A, B, F, I, and J). Importantly, we identified a novel immune evasion mechanism whereby SVV utilizes its 3C protease to specifically target and degrade Nrf2 in a caspase-dependent manner (Fig. 3A through H), thereby subverting this critical host defense pathway to facilitate viral persistence and pathogenesis. These results collectively establish Nrf2 as a central regulator of host antiviral responses and reveal a sophisticated viral countermeasure to overcome cellular antioxidant defenses.

During PRRSV infection, HO-1 serves as a key antiviral component (38). Xanthohumol, a natural compound, inhibits PRRSV proliferation and alleviates virus-induced oxidative stress by activating the Nrf2/HO-1 signaling axis (63). Artesunate, an antimalaria drug, suppresses the replication of PRRSV through the activation of AMPK and Nrf2/HO-1 signaling cascades (64). PRRSV-induced HOXA3 suppresses the IFN-I response by negatively regulating the transcription of HO-1 (65). Carbon monoxide suppresses the replication of PRRSV via the cyclic GMP/protein kinase G and NF-κB signaling pathway (48). Notably, biliverdin—a metabolic product of HO-1, not iron—is the critical effector that hinders PRRSV replication (66). Conversely, microRNAs miR-22 and miR-24-3p facilitate viral replication by post-transcriptionally repressing HO-1 expression (67, 68). Mechanistically, the viral nsp5 protein disrupts Nrf2/HO-1 pathway activation by targeting p62, thereby antagonizing the antiviral functions of this cytoprotective axis (69). In this study, we demonstrate that pharmacological activation of HO-1 using CoPP or hemin, as well as HO-1 overexpression, significantly suppressed SVV replication while promoting p62 accumulation (Fig. 2K, M, 5A through C and G through I). Conversely, HO-1 inhibition with ZnPP or siRNA-mediated knockdown enhanced viral replication (Fig. 2J, L, and 5I). Treatment with CORM-2 (a CO-releasing molecule) or biliverdin produced effects similar to HO-1 activation, suggesting that HO-1 metabolites (CO and biliverdin) play crucial roles in restricting SVV replication (Fig. 6A through G). Furthermore, CORM-2 treatment effectively inhibited SVV-induced NF-κB signaling and inflammatory cytokine production (Fig. 6H through I). Furthermore, SVV takes advantage of the DNA damage response (DDR) as a means to support its own replication. Concurrently, the DDR triggered by SVV initiates NF-κB signaling, with a subsequent upregulation in the levels of pro-inflammatory cytokines (70). Inhibiting ferroptosis not only significantly reduces SVV replication efficiency but also alleviates its associated inflammatory effects (71). Notably, both SVV infection and transfection with the 3C protein alone could downregulate HO-1 protein expression, revealing a viral strategy to counteract this host defense mechanism.

HSV-1-induced Nrf2 degradation contributes to ferroptosis and is associated with viral encephalitis (30). SARS-CoV-2 ORF3a degrades Nrf2 by recruiting Keap1-promoted cells to ferroptosis (34). Dengue virus NS2B3 targets Nrf2 for degradation, resulting in increased oxidative stress and viral replication (32). Nrf2 agonists and inhibitors were used to investigate the role of Nrf2 in SVV infection. Similar to TBHQ, 4-OI and DMF are Nrf2 agonists that induce cellular antiviral activity to inhibit the replication of SARS-CoV2, HSV-1 and HSV-2, vaccinia virus, and Zika virus (ZIKV) (41). 4-OI and DMF reduced the inflammatory response to SARS-CoV2 infection, and treatment with DMF decreased the mRNA levels of IFNB1, CXCL10, and CCL5 (41). Treatment with 4-OI significantly increased the expression of HO-1, NQO1, and p62 (58). Consistent with previous studies, we found that HO-1 and p62 expression was upregulated with TBHQ (Fig. 4D) while limiting the inflammatory response (Fig. 4H and J).

HO-1 is a stress-inducible enzyme that converts heme into CO, BV, and iron (72). The anti-inflammatory and antioxidant effects of HO-1 aid cellular homeostasis (72). HO-1 is a critical mediator of the IFN-I signaling pathway that inhibits viral replication (43, 44). The HO-1 agonist, CoPP, inhibits influenza and dengue virus replication by inducing an IFN-I response (43, 73). Hemin shows the ability to act as an antiviral agent against hepatitis A virus, Ebola virus, ZIKV, PRRSV, and HIV (36, 46, 47, 74, 75). Hemin defends against ZIKV invasion by interrupting the fusion between the virus and the endosome (76). Hemin’s ability to inhibit the replication of PRRSV depends significantly on HO-1 and is not dependent on iron (75). The metabolic products of HO-1, CO, and BV inhibit PCV3 replication. CO acts through the CO-dependent cGMP/PKG pathway; BV acts either through the BV/BR/NO-dependent cGMP/PKG pathway or by reducing ROS. However, iron, the third metabolic product, has no such inhibitory effect on PCV3 replication (37). We found that CoPP inhibited SVV replication by upregulating HO-1 expression (Fig. 5A through C).

HO-1 metabolites CO and BV exert antiviral activity. CORM-2, a CO inducer, inhibits PRRSV and PCV3 replication by activating cyclic GMP/protein kinase G and negatively regulating the NF-κB signaling pathway (37, 48). Similar to CO, CORM-2 promotes HO-1 expression in a positive feedback loop, which plays a role in anti-PCV3 infection (37). In this study, we found that CORM-2 and BV inhibited SVV infection (Fig. 6G). CORM-2 attenuated SVV-induced NF-κB activation and inflammatory factor expression (Fig. 6H through I). The core end products of the Nrf2/HO-1 pathway are primarily bilirubin, CO, and free iron ions (Fe²^+^), which are generated by the degradation of heme catalyzed by HO-1. Existing studies have clearly demonstrated that these substances can exert feedback regulation on Nrf2. Bilirubin, with its antioxidant properties, helps maintain cellular redox homeostasis, which can indirectly support Nrf2 activation by reducing oxidative stress (77). CO mediates HO-1 induction via Nrf2 activation (78). NAC suppresses Coxsackievirus B type 3 replication and inflammatory response to minimize myocardial injury (79). NAC exerts anti-PRRSV by decreasing LC3-II expression and inhibiting p62 degradation (80). We found that NAC severely inhibited LC3-II conversion and inflammatory factor transcription (Fig. 7A through G).

HO-1 plays a dual part in regulating antiviral immune reactions and alleviating excessive inflammatory harm during influenza virus invasion (81). HO-1 was a key negative regulator of radiotherapy-induced IFN-I signaling. HO-1 inhibited IFN-I production by comprehensively disturbing the distribution of cGAS and STING during radiotherapy, independent of its enzymatic activity (82). Moreover, studies have indicated that HO-1 interacts with IRF-3 to form a complex. This complex enables the nuclear translocation of IRF-3 and enhances the expression of IFN-α/IFN-β, thus triggering the antiviral immune response (43, 83, 84). Hyperoside inhibits the infection of equine herpesvirus type 8 via reducing oxidative stress and IFN-I synthesis, which is accomplished by triggering the JNK/Keap1/Nrf2/HO-1 signaling cascades (45). p21 inhibits influenza A virus through disrupting the viral polymerase complex and enhancing IFN-I signaling; p21 contributes to the activation of IRF-3 through impeding the K48-linked ubiquitination degradation process of HO-1, thereby enhancing the expression of IFN-I (64). HO-1 was crucial for its antiviral function through IFN-I. During carp spring viremia virus (SVCV) infection, HO-1 was capable of enhancing the production of IFN-I, and at the same time, it could reduce the replication of SVCV (85). HO-1 product BV appears to disrupt the oxidative stress caused by hepatitis C virus (HCV) replication by triggering the expression of antiviral interferon, including interferon alpha2 and alpha17 (86). BV powerfully inhibits the HCV NS3/4A protease, and this inhibition probably accounts for the antiviral function of HO-1 (87). HO-1 boosts the number of macrophages, strengthening antiviral responses through the IFN pathways. In line with this, HO-1 knockout mice suffer from more severe infections, and HO-1 recruitment leads to the inhibition of IAV replication and the alleviation of pulmonary inflammation (81). CoPP, an HO-1 agonist, curbs IAV replication through the IRF-3-mediated synthesis of IFN-α/IFN-β (43). We found that ectopic expression of HO-1, HO-1 agonist CoPP, and HO-1 product biliverdin significantly elevated the levels of IFN-I and ISGs (Fig. 8A through L). Biliverdin interferes with HCV replication-mediated oxidative stress by inducing expression of antiviral interferons, such as interferon alpha2 and alpha17; biliverdin treatment also induces expression of OAS1/2/3 and PKR (86). Knockdown of HO-1 partially dampened the rises in IFN-β promoter activity and ISRE promoter activity induced by poly(I:C) (Fig. 8J and K). Meanwhile, the levels of IFN-I and ISGs attenuated after depletion of HO-1 (Fig. 8L). These findings suggest that HO-1 enhances the IFN-I response and stimulates the expression of ISGs, thereby contributing to its antiviral function.

Overall, our study proposes a model in which SVV infection induces ROS accumulation and GSH depletion. SVV 3C^pro^ targets the Nrf2/HO-1 oxidative stress axis for degradation to facilitate viral replication. HO-1 facilitates the IFN-I response and ISG expressions, contributing to its antiviral mechanism. These results offer further understanding of the pathogenesis of SVV, thereby contributing to the identification of a possible target for intervening in oxidative stress during SVV infection.

## MATERIALS AND METHODS

### Cells, viruses, antibodies, and reagents

BHK-21 cells (ATCC, CCL-10), PK-15 cells (ATCC, CCL-33), and HEK-293T cells (ATCC, CRL-11268) were grown in Dulbecco’s modified Eagle’s medium (Invitrogen, California, USA) containing 10% fetal bovine serum (FBS) (Invitrogen) at 37°C with 5% CO_2_. The SVV strain CHhb17 was used in our previous studies (88). eGFP-tagged recombinant SVV was kindly provided by Fuxiao Liu (Qingdao Agricultural University) (89). Rabbit anti-Nrf2 (80593-1-RR), rabbit anti-HO-1 (10701-1-AP), rabbit anti-Keap1 (10503-2-AP), rabbit anti-NQO1 (11451-1-AP), rabbit anti-caspase3 (19677-1-AP), rabbit anti-GFP (50430-2-AP), rabbit anti-ISG56 (23247-1-AP), rabbit anti-ISG54 (12604-1-AP), rabbit anti-OAS3 (21915-1-AP), and rabbit anti-Mx1 (13750-1-AP) were purchased from Proteintech (Wuhan, China). Mouse anti-HA tag monoclonal antibody (A1933) was purchased from Abclonal (Wuhan, China). LC3B (L7543) was purchased from Sigma-Aldrich. SQSTM1/p62 (ab56416, Abcam), p-AMPK (2535, Thr172; Cell Signaling Technology), HRP-conjugated goat antirabbit IgG (H+L) (1706515, Bio-Rad), HRP-conjugated goat antimouse IgG (H+L) (1706516, Bio-Rad), FITC-conjugated goat antimouse IgG (H+L) (F0257, Sigma), and Alexa-568-conjugated goat antirabbit IgG (H+L) (11011, Invitrogen) was purchased from Invitrogen. Mouse anti-VP1 monoclonal antibody has been used in our previous studies (88). TBHQ (HY-100489), ML385 (HY-100523), Cobalt protoporphyrin IX (CoPP, HY-W250116), ZnPP (HY-101193), CORM-2 (HY-W033577), BV (HY-135005A), NAC (HY-B0215), bilirubin (HY-N0323, BR), ODQ (HY-101255, an sGC inhibitor), and KT5823 (HY-N6791, a PKG inhibitor) were obtained from MedChemExpress (Shanghai, China). MG132 (S2619), Z-VAD-FMK (S7023), bafilomycin A1 (S1413), CQ (S6999), and 3-MA (S2767) were purchased from Selleck Chemicals (Shanghai, China).

### Plasmid construction

Nrf2 and HO-1 genes were amplified from BHK-21 cells and ligated into pCMV-HA vectors (Clontech, 631604) by using DNA assembly mix plus (D0204P; Lablead, China). GFP-tagged SVV structural and non-structural protein plasmids, single point mutant plasmids GFP-3C^H48A^ and GFP-3C^C160A^, and double mutant plasmid GFP-3C^[DM]^ (H48A and C160A double mutants) that have been used in our previous studies (11).

### Detection of intracellular ROS levels

Intracellular ROS was detected with DCFH-DA fluorescent probe (S0033S; Beyotime, Shanghai, China). BHK-21 cells were stained with DCFH-DA fluorescent probe for 30 min at 37°C in a cell culture incubator. After two washes with phosphate-buffered saline (PBS), BHK-21 cells were incubated with new culture medium and captured under a Nikon A1 confocal microscope. For flow cytometry analysis, washed cells were collected by trypsinization. After centrifugation at 300 × *g* for 5 min, the cell pellets were resuspended in PBS and analyzed using a NovoCyte flow cytometer (Agilent, California, USA).

### Measurement of MDA and GSH levels

MDA and GSH were assessed using specific assay kits. BHK-21 cells were seeded in six-well plates. MDA concentrations were tested with an MDA assay kit (S0131M, Beyotime) according to the manufacturer’s instructions. The absorbance at 532 nm was measured using a plate reader (Bio-Rad). GSH content was examined by reduced glutathione content assay kit (BC1175; Solarbio, China). The absorbance at 532 nm was measured using a plate reader (Bio-Rad).

### Western blot

Following sample collection, cells were lysed with gentle rotation at 4°C for 30 min and subsequently centrifuged at 13,000 × *g* (4°C) for 15 min to remove cellular debris. The resulting supernatant was mixed with loading buffer and heat-denatured at 95°C for 10 min. Proteins were then resolved by sodium dodecyl sulfate-polyacrylamide gel electrophoresis and electrophoretically transferred onto nitrocellulose membranes (66485; Pall, Florida, USA). For immunoblotting, membranes were first blocked with 5% skim milk in PBST for 1 h at room temperature (RT), followed by overnight incubation with primary antibodies at 4°C. After thorough PBS washing, membranes were incubated with appropriate HRP-conjugated secondary antibodies for 2 h at RT. Finally, after additional PBS washes, protein signals were detected using enhanced chemiluminescence reagent (E1070, Lablead).

### Quantitative RT-PCR

Total RNA was isolated using the FastPure Cell/Tissue Total RNA Isolation Kit (Vazyme, RC101-01), and cDNA synthesis was performed using 1 µg of total RNA with the First-Strand Synthesis Master Mix (Lablead, F0202). Quantitative RT-PCR was then carried out with the resulting cDNA using Realab Green PCR Mix (Lablead, R0202) on a Bio-Rad CF96 Real-Time PCR System. Data were normalized to the β-actin level of each sample; the relative gene expression was determined by calculating 2^−ΔΔCT^.

### Indirect immunofluorescence

Cells grown on sterile coverslips in 24-well plates were fixed with freshly prepared 4% paraformaldehyde for 10 min at RT. Following three PBS washes, cells were permeabilized with 0.1% Triton X-100 in PBS containing 2% bovine serum albumin (BSA) for 10 min at RT. Non-specific binding sites were then blocked with 2% BSA in PBS for 30 min at RT. Samples were incubated with primary antibodies diluted in blocking buffer overnight at 4°C. After extensive PBS washing, appropriate secondary antibodies were applied for 1 h at RT in the dark. Following final PBS washes, the cells were incubated with DAPI for 5 min for nuclear staining. Fluorescence images were acquired using a Nikon A1 confocal laser scanning microscope (Nikon, Tokyo, Japan).

### Nuclear and cytoplasmic fractionation

SVV-infected BHK-21 cells were collected at indicated times post-infection, then subjected to nuclear and cytoplasmic fractionation using a nuclear and cytoplasmic fractionation kit (78833; Thermo Fisher, USA) following the manufacturer’s instructions. The nuclear and cytoplasmic fractions were examined using Western blot.

### RNA interference

The siRNAs were devised and fabricated by GenePharma (Suzhou, China): si-Nrf2-196 (sense, 5′-GGAUUUGAUUGACAUCCUUTT-3′; antisense, 5′-AAGGAUGUCAAUCAAAUCCTT-3′), si-Nrf2-1313 (sense, 5′-GGUCCUAAAGGACAGCCAATT-3′; antisense, 5′-UUGGCUGUCCUUUAGGACCTT-3′), si-HO-1-189 (sense, 5′-GGAGGAGAUAGAACGCAAUTT-3′; antisense, 5′-AUUGCGUUCUAUCUCCUCCTT-3′), si-HO-1-620 (sense, 5′-CAUUCCUGCUCAACAUUGATT-3′; antisense, 5′-UCAAUGUUGAGCAGGAAUGTT-3′), si-Caspase-3 (sense, 5′-GGGUGUGUGUAUAAUAAUUTT-3′; antisense, 5′-AAUUAUUAUACACACACCCTT-3′), and siNC (sense, 5′-UUCUCCGAACGUGUCACGUTT-3′; antisense, 5′-ACGUGACACGUUCGGAGAATT-3′) served as a control. BHK-21 cells in six-well plates were transfected with siRNAs using Lipofectamine RNAiMAX (13778150, Thermo Fisher). Cells were infected with SVV at 36 h post-transfection, and the expression of proteins was subjected to Western blot. Lipofectamine RNAiMAX (13778150, Thermo Fisher) was used to transfect siRNAs into BHK-21 cells seeded in six-well plates. At 36 h after transfection, the cells were infected with SVV, and the protein expression was analyzed by Western blot.

### Viral infection and titration

BHK-21 cells were infected with SVV at the indicated multiplicities of infection (MOI). Following a 1 h adsorption period at 37°C, cells were washed three times with DMEM to remove unbound virus and then maintained in fresh medium containing 2% FBS. At designated timepoints post-infection, both culture supernatants and cell lysates were harvested. Viral titers were determined by a limiting dilution assay on fresh BHK-21 monolayers, with infectivity quantified using the TCID_50_ method according to Reed and Muench (88).

### GST pull-down

Recombinant GST-3C and His-Nrf2 or HO-1 (His-HO-1) proteins were expressed in *Escherichia coli* BL21 (DE3) and purified using glutathione-Sepharose 4B beads (GE Healthcare) and Ni-NTA agarose (Qiagen), respectively.

For GST pull-down assays, purified GST-3C or GST alone was immobilized on glutathione-Sepharose 4B beads at 4°C for 1 h with gentle rotation. After washing the beads three times, equal amounts of His-Nrf2 or His-HO-1 were added to the bead-protein mixture and incubated at 4°C overnight with gentle rotation. The beads were then washed five times with washing buffer (50 mM Tris-HCl, pH 7.5, 300 mM NaCl, 1 mM EDTA, 0.5% Triton X-100). The eluted proteins were detected by Western blot.

### Luciferase reporter assay

HEK-293T cells were seeded in 24-well plates and co-transfected with the specified plasmids, along with IFN-β reporter plasmid, ISRE reporter plasmid, and the pRL-TK plasmid. At 24 h post-transfection, the luciferase activities of Firefly and Renilla were measured using the dual-luciferase reporter assay kit (Promega). The measurements were carried out strictly following the manufacturer’s protocol, with Renilla luciferase activity serving as an internal control. Every experiment was repeated independently three times.

### Statistical analysis

Statistical analysis was performed using GraphPad Prism software (GraphPad Software, San Diego, CA, USA). Statistical significance was evaluated by using one-way analysis of variance. Data from three biologically independent experiments are presented as mean ± standard deviation. Statistical significance was defined as follows: not significant for *P* ≥ 0.05; *P < 0.05, **P < 0.01, and ***P < 0.001 were considered statistically significant.

## Data Availability

The data supporting this study’s findings are available from the corresponding author upon reasonable request.
